# All hands on deck: early graduation of senior medical students in the COVID-19 pandemic

**DOI:** 10.15694/mep.2020.000096.1

**Published:** 2020-05-13

**Authors:** Laith Alexander, James Ashcroft, Matthew H. V. Byrne, Jonathan Wan

**Affiliations:** 1 School of Clinical Medicine; 2 Cambridge University Hospitals Trust

**Keywords:** Medical students, COVID-19, graduation, junior doctors

## Abstract

This article was migrated. The article was marked as recommended.

During the COVID-19 pandemic, early graduation of senior medical students simultaneously offers useful clinical experience in preparation for junior doctor posts, whilst helping address staffing shortages due to illness or self-isolation. Having recently graduated early from medical school, we offer our reflections on the obstacles and opportunities associated with working in an uncharted clinical environment. We are not the only ones on a steep learning curve at this time: this pandemic will challenge and provide learning for staff of all levels.

## Introduction

The spread of COVID-19 is putting extreme pressure on healthcare systems globally. To bolster the healthcare workforce at this unprecedented time, clinical academics and recently retired doctors have been brought back into clinical practice ( Haynes, 2020), clinical rotas have been re-organised, and final-year medical students who have been deemed to have reached their competencies are graduating early. Final-year medical students in Italy, USA and now the UK can register to work and apply their skills early to assist in the COVID-19 pandemic ( Harvey, 2020).

In the UK, senior medical students are being offered the opportunity to begin their post-graduate training early, involving provisional registration and interim contracts with similar terms to junior doctor contracts ( UK Foundation Programme, 2020). To protect new doctors, the contracts stipulate several safeguards, including assignment of a named clinical supervisor; direct supervision when seeing acutely unwell patients, and daytime working hours. It is expected that new doctors would be deployed in supporting areas, avoiding COVID-19 escalation wards and intensive care units where possible.

## Reflection

Having recently graduated early from medical school, we offer our own reflections on the obstacles and opportunities associated with working in an uncharted clinical environment. We know that we will be joining the workforce at a challenging time. Beyond the direct threat that COVID-19 may pose to our own health, we are concerned that staff sickness and self-isolation may have substantial effects on our supervision ( Hope, 2020). Clinicians will be in a state of rapid flux and turnover, and at a time where we would already be adapting to new environments and new people which may become disorienting. The importance of adequate supervision and rapport building with our mentors remains constant but adapting to these challenges requires flexibility within the clinical team. How we respond to the pandemic may set a precedent for how we manage the medical student to junior doctor transition in the future.

Reassuringly, The Medical Schools Council of the UK has stated that medical students working pre-qualification should not be allowed to work beyond their competencies and that shadowing and induction must continue as normal ( Medical Schools Council, 2020). We recognise that challenging clinical situations - such as exposure to difficult decision making and end of life scenarios - may approach earlier in our careers than previously anticipated. We hope that we can seek senior support when faced with challenging situations that stretch or fall outside of our skillset. Needless to say, proper supervision will reduce medical errors, but if mistakes do occur, we need senior support for reflection and to derive learning benefits which could benefit the whole team ( Kroll *et al.*, 2008).

In a time where all doctors are adapting to a new way of working, it should not be assumed that we are aware of pre-existing protocols. Nowhere is this more apparent than in the case of personal protective equipment (PPE). We are fortunate to have had recent online training how to don and doff PPE correctly, though local hospital inductions should ensure that all staff are performing this important procedure to a high standard.

The pandemic reflects an opportunity for immersion of senior medical students in a clinical team. The communities of practice theory of medical education highlights learning-by-participation rather than learning-by-acquisition, where the currency of learning is authentic work ( Morris, 2018). Never has the opportunity been greater for senior medical students to learn by becoming a core part of the professional community. We hope that our learning won’t only be semantic: it should include management, personal skills and the fostering of an appreciation for the importance of the wider multidisciplinary clinical team, including allied health professionals, in our response to the pandemic. Furthermore, the disruption to the ‘social hierarchy’ of the clinical team caused by this novel challenge may benefit learning, as power dynamics are known to inhibit full engagement with the clinical team ( Pemberton, Mavin and Stalker, 2007).

The pandemic should not be a time of educational stasis, it is a chance to harness the power of technology and novel educational tools. Digital inductions and virtual webinars with educational supervisors are one possibility, and medical schools are adopting this widely to deliver final sets of lectures and even high-stakes exams. These steps are necessary now but could also be used to supplement invaluable face-to-face contact in the future.

Whilst many medical students may feel compelled to work, we think it is important to be mindful of the risk of burnout inherent in entering a stressful job following premature termination of medical school with minimal interleaving holiday. Some steps can be put in place now to minimise risk. For example, it is important that starting work early is voluntary; we are pleased that this is the case for us in the UK. For some students, the time between ending medical school and starting work will be needed as a valuable break.

Lastly, the support networks normally available to junior doctors must still be present for senior medical students as they transition, albeit in a different format given social distancing. Again, technology can be leveraged: for example, with regular video calls using a ‘Schwartz Round’ format, or instant messaging groups. In the coming months, an open culture of talking about concerns, mental wellbeing and experiences on the wards must be fostered, recognising that being mindful, open and honest about your concerns is a crucial part of doctors’ development. As final year students, we hope that these opportunities will be available to us, so we can take the lessons learnt with us in our future career.

## Conclusion

As medical students transitioning to our new roles as doctors, we hope to bring with us enthusiasm, passion and a desire to innovate. We see the obstacles posed by COVID-19 equally as opportunities for mentorship, collaboration and mutual learning. We are open to the lessons from our senior clinicians, and we hope they are open to our perspectives too. In a situation where even the most senior clinicians are apprentices, the whole profession can grow from the challenges we face.

## Take Home Messages

• As medical students transitioning to our new roles as doctors, we hope to bring with us enthusiasm, passion and a desire to innovate.
• How medical educators respond to the pandemic may set a precedent for how we manage the medical student to junior doctor transition in the future.
• We hope that we can seek senior support when faced with challenging situations that fall outside of our skillset.
• The pandemic offers a chance to harness the power of technology for education.
• Never has the opportunity been greater for senior medical students to learn by becoming a core part of the professional community.

## Notes On Contributors

Laith Alexander is an MB/PhD student at the University of Cambridge. His interests lie in general practice, public health and psychiatry. His PhD in Neuroscience explored the role of the ventromedial prefrontal cortex in the regulation of emotion and cardiovascular function. He is due to start an Academic Foundation Programme junior doctor post in Psychological Medicine & Psychiatry at St Thomas’ Hospital, London in August 2020. ORCID: https://orcid.org/0000-0003-1297-6548

Matthew H. V. Byrne is an anatomy demonstrator at the University of Cambridge and will start as an Academic Clinical Fellow in Urology at Oxford in August. He is currently undertaking a PGCERT in medical education at the University of Cambridge and has previously be awarded a MRes in Transplantation. He founded a national charity that facilitates medical student volunteers delivering talks at secondary schools. ORCID: https://orcid.org/0000-0002-2414-352X

James Ashcroft is an Academic Clinical Fellow in General Surgery in the East of England Deanery with an interest in early academic education and technical performance training. His educational work has explored cognitive interventions in technical skill (awarded MRes) and early surgical learning (awarded MSc).

Jonathan C. M. Wan is an MB/PhD student at Trinity College, University of Cambridge. He carried out his PhD in computational cancer diagnostics and is continuing his research as a Bioinformatics Engineer at Memorial Sloan Kettering Cancer Center. He will be commencing an Academic Foundation Programme post in Oncology at Guy’s and St Thomas’ from August 2020. He is interested in oncology research and medical education. ORCID: https://orcid.org/0000-0003-0001-1802
