# Supplementary material for: All hands on deck: early graduation of senior medical students in the COVID-19 pandemic
Source: MedEdPublish (2016). 2020 May 13;9:96. [Version 1] doi: 10.15694/mep.2020.000096.1 (PMC10697452; doi:10.15694/mep.2020.000096.1)
Supplement: Peer review history

# Reviewer report: Reviewer response for version 1

**DOI:** 10.21956/mep.20086.r30931

**Published:** 2020-08-30

**Authors:** Ronald M Harden

**Affiliations:** 1 AMEE

This review has been migrated. The reviewer awarded 4 stars out of 5 This article is to be welcomed given that students are acknowledged now as partners in the learning process. I recommend the article as a useful read for all with an interest in medical education. Three things struck me about the article. 1.The suggested issues to be addressed if clinical experience is provided. 2.The argument that the pandemic should not be a time for educational stasis and 3. The plea that in the future more emphasis should be placed in medical education on immersing students as part of a clinical team

Reviewer Expertise:

NA

No decision status is available

# Reviewer report: Reviewer response for version 1

**DOI:** 10.21956/mep.20086.r30928

**Published:** 2020-05-14

**Authors:** David Bruce

**Affiliations:** 1 None

This review has been migrated. The reviewer awarded 4 stars out of 5 In this paper the authors lay out their apprehensions and hopes as they start their medical careers early as interim Foundation Year 1 doctors (FiY1). Many good points are made and their views will be interesting to new doctors in a similar position as well as senior doctors in the medical teams where they will work. While some of the concerns voiced will be generic to all new doctors, the COVOD pandemic has created specific issues and requirements – such as the need for PPE training and changes to normal support systems as a result of social distancing and it was helpful to hear the perspectives of those transitioning from senior medical students to new FiY1 doctors.The important matter of doctor wellbeing is also discussed. Again – the early start to working life without time for a break or holiday may be a cause of stress for some. As well as the importance to the safety and wellbeing of the doctors, we know that stress in medical teams can result in a decrease it the safety and quality of patient care. The work by Coia and West emphasises the importance of the autonomy, belonging and competence as key needs for wellbeing and motivation at work. Suggestions for improved use of technology in promoting learning are helpful and Schwartz Rounds are certainly worth exploring.The paper is well written, and I would only make two comments that might improve the authors message. First – I was unclear what was meant by the authors’ hope that learning wouldn’t only be semantic, and second, I felt the reference to senior medical students was confusing as they had graduated and were provisionally registered with the GMC – so new doctors might be better. Finally – as the GMC has stated “The placement of these new provisionally registered interim Foundation Year 1 doctors will be facilitated by medical schools working with foundation schools and in support of service providers. Deploying these doctors will take account of the need for induction, supervision, and support for their wellbeing, and into roles appropriate to their skill set and that best support delivery of frontline services.” It will be valuable to have a follow up paper that describes the experiences of the authors having completed this new start to their medical careers.

Reviewer Expertise:

NA

No decision status is available

# Reviewer report: Reviewer response for version 1

**DOI:** 10.21956/mep.20086.r30929

**Published:** 2020-05-14

**Authors:** P Ravi Shankar

**Affiliations:** 1 American International Medical University

This review has been migrated. The reviewer awarded 3 stars out of 5 This is another of a series of articles on the COVID-19 pandemic and its impact on education written from the perspective of senior medical students. The authors can briefly describe how they are graduating early and modifications to the usual graduation process due to the pandemic. They have mentioned their apprehensions and concerns some of which have also been addressed in other articles. The pandemic offers both challenges and opportunities. The creation of a supported environment is important. I am not sure when the authors will enter the clinical workforce or whether they have already started working. Many of their concerns may also be applicable to situations other than the COVID-19 pandemic.

Reviewer Expertise:

NA

No decision status is available

# Reviewer report: Reviewer response for version 1

**DOI:** 10.21956/mep.20086.r30930

**Published:** 2020-05-13

**Authors:** James Gray

**Affiliations:** 1 University of Sheffield

This review has been migrated. The reviewer awarded 2 stars out of 5 An interesting reflective piece to read however given that this is written pre-commencement of the posts it is difficult to know whether this is truly relevant to Covid19 or simply a reflection of all the concerns and insecurities of the transition. On that basis it is a potentially interesting read for all those who will welcome newly qualified clinicians into their teams however I don't think it necessarily adds a great deal that is Covid specific. A more valuable piece of work would be to evaluate these thoughts against the reality after a period in post as this may lead to valuable insights to aid supporting the transition from student to clinician in the future with possible innovations that have come out of this pandemic being shown to add value.

Reviewer Expertise:

NA

No decision status is available
